# Supplementary material for: Light Quality Impacts Vertical Growth Rate, Phytochemical Yield and Cannabinoid Production Efficiency in Cannabis sativa
Source: Plants (Basel). 2022 Nov 4;11(21):2982. doi: 10.3390/plants11212982 (PMC9659141; doi:10.3390/plants11212982)
Supplement: Supplementary file 1 [file plants-11-02982-s001.zip › plants-1956709-supplementary.pdf]

Supplementary Table S1. Significant difference between replicates of average environmental parameters and plant measurements. Values are shown as mean ± SD. T-tests assume unequal variances where df stands for degrees of freedom using first order Kenward and Roger correction. Avg. stands for average, veg. for vegetative, flo. for flowering, ss for statistical significance, ns for non-significant. Asterisks denote statistically significant p-value where * is for p <0.05, ** is for p<0.01, *** is for p<0.0001.

Plant average	Replicate 1	Replicate 2	df	t Ratio	p	ss
**Starting height (cm)**	15.5±2.3	8.7±1.2	93.05	-4.706	<0.0001	***
**Final height (cm)**	76.1±7.3	70.4±5	79.35	-19.292	<0.0001	***
**PPFD (umol m² s⁻¹)**	421.1±4.1	392.4±3.0	96.3	-5.673	<0.0001	***
**Fresh inflorescence (g)**	116.2±25.5	104.5±28.8	104.5	-2.245	<0.05	*
**Dry inflorescence (g)**	23.3±5.3	19.4±5.8	105.4	-3.685	<0.0005	**
**Avg. veg. growth rate (cm week⁻¹)**	7.8±1.5	7.5±1.9	100.4	-1.0159	0.3121	ns
**Avg. flo. growth rate (cm week⁻¹)**	15.0±2.1	15.6±1.2	85.9	1.635	0.1057	ns
**Total THC concentration(%)**	7.11±1.94	9.18±1.08	26.577	3.977	<0.0005	***
**Total CBD concentration(%)**	4.95±1.08	5.87±0.67	28.556	3.047	<0.005	**
**Total CBG concentration(%)**	0.08±0.05	0.12±0.05	33.954	2.003	0.0532	ns
**Total terpene concentration(mg g⁻¹)**	23.27±6.32	25.62±3.69	27.372	1.364	0.1838	ns
**Total THC yield (g plant⁻¹)**	1.65±0.51	1.87±0.7	31.12528	1.087942	0.285	ns
**Total CBD yield (g plant⁻¹)**	1.16±0.36	1.21±0.5	30.95745	0.338538	0.7372	ns
**Total CBG yield (g plant⁻¹)**	0.02±0.01	0.02±0.01	33.35843	1.439915	0.1592	ns
**Total terpene yield (mg plant⁻¹)**	476.6±169.7	595.8±215.9	32.20433	1.841671	0.0747	ns

Supplementary Table S2. Concentrations of 80 terpene compounds in *C. sativa* plants cultivated under different light treatments. Different letters represent significant difference between treatments by Tukey HSD (p<0.05). ''ND'' stands for ''not detected''.

Terpene Compound (mg g⁻¹)	HPS	Amber	Red	Rose	Purple	Blue
Myrcene	7.504 ±0.43^{ab}	4.809 ±0.44^{c}	5.669 ±0.757^{bc}	7.347 ±0.573^{ab}	8.914 ±0.53^{a}	7.898 ±0.653^{a}
α-Pinene	5.339 ±0.458	4.488 ±0.256	4.738 ±0.129	5.138 ±0.317	5.715 ±0.367	5.733 ±0.385
Limonene	2.666 ±0.122^{ab}	1.857 ±0.162^{c}	2.178 ±0.246^{bc}	2.594 ±0.177^{abc}	3.051 ±0.119^{a}	2.656 ±0.23^{ab}
β-Pinene	2.474 ±0.162^{ab}	1.929 ±0.106^{b}	2.03 ±0.116^{b}	2.3 ±0.082^{ab}	2.708 ±0.136^{a}	2.687 ±0.16^{a}

Terpene Compound (mg g^{-1})	HPS	Amber	Red	Rose	Purple	Blue
Linalool	0.717 ±0.065^{ab}	0.428 ±0.067^{b}	0.467 ±0.097^{ab}	0.59 ±0.068^{ab}	0.847 ±0.045^{a}	0.845 ±0.02^{a}
(E)-β-Ocimene	0.596 ±0.056^{abc}	0.347 ±0.039^{c}	0.437 ±0.066^{bc}	0.625 ±0.069^{ab}	0.75 ±0.066^{a}	0.631 ±0.053^{ab}
endo-Fenchol	0.402 ±0.026	0.298 ±0.046	0.298 ±0.052	0.373 ±0.041	0.458 ±0.025	0.46 ±0.012
β-Caryophyllene	0.396 ±0.031	0.233 ±0.028	0.275 ±0.046	0.371 ±0.041	0.413 ±0.039	0.377 ±0.076
Selina-4(15),7(11)-diene	0.363 ±0.031^{abc}	0.217 ±0.024^{c}	0.26 ±0.042^{bc}	0.323 ±0.04^{abc}	0.414 ±0.052^{ab}	0.466 ±0.021^{a}
Selina-3,7(11)-diene	0.487 ±0.038^{ab}	0.284 ±0.032^{c}	0.336 ±0.053^{bc}	0.43 ±0.053^{abc}	0.556 ±0.058^{a}	0.614 ±0.026^{a}
Guaiol	0.344 ±0.04^{ab}	0.189 ±0.029^{b}	0.21 ±0.043^{b}	0.293 ±0.043^{ab}	0.39 ±0.056^{a}	0.454 ±0.031^{a}
10-epi-γ-Eudesmol	0.327 ±0.066^{ab}	0.193 ±0.032^{b}	0.217 ±0.048^{b}	0.3 ±0.05^{ab}	0.397 ±0.064^{ab}	0.456 ±0.027^{a}
Bulnesol	0.375 ±0.05^{ab}	0.213 ±0.039^{b}	0.24 ±0.055^{b}	0.32 ±0.057^{ab}	0.408 ±0.056^{ab}	0.492 ±0.048^{a}
trans-Pinene hydrate	0.292 ±0.024	0.208 ±0.036	0.211 ±0.04	0.266 ±0.032	0.324 ±0.022	0.329 ±0.012
α-Terpineol	0.38 ±0.032	0.275 ±0.046	0.283 ±0.054	0.343 ±0.041	0.426 ±0.03	0.434 ±0.014
Germacrene B	0.232 ±0.032^{abc}	0.105 ±0.019^{c}	0.121 ±0.03^{c}	0.162 ±0.028^{bc}	0.258 ±0.04^{ab}	0.322 ±0.032^{a}
Citronellol	0.174 ±0.014^{ab}	0.124 ±0.022^{b}	0.129 ±0.027^{ab}	0.151 ±0.014^{b}	0.219 ±0.012^{a}	0.233 ±0.01^{a}

Terpene Compound (mg g^{-1})	HPS	Amber	Red	Rose	Purple	Blue
α-Eudesmol	0.209 ±0.027^{ab}	0.118 ±0.021^{b}	0.118 ±0.034^{b}	0.172 ±0.03^{ab}	0.237 ±0.037^{ab}	0.284 ±0.017^{a}
β-Eudesmol	0.178 ±0.022^{abc}	0.095 ±0.016^{c}	0.107 ±0.023^{bc}	0.144 ±0.024^{abc}	0.201 ±0.03^{ab}	0.237 ±0.015^{a}
γ-Eudesmol	0.067 ±0.007^{a}	0.023 ±0.007^{b}	0.035 ±0.007^{ab}	0.046 ±0.008^{ab}	0.074 ±0.009^{a}	0.066 ±0.01^{a}
α-Bisabolol	0.12 ±0.01	0.069 ±0.008	0.093 ±0.017	0.109 ±0.013	0.116 ±0.017	0.117 ±0.011
Borneol	0.136 ±0.006	0.1 ±0.012	0.106 ±0.015	0.121 ±0.008	0.148 ±0.004	0.147 ±0.005
Hexanol	0.017 ±0.005	0.01 ±0.001	0.013 ±0.003	0.022 ±0.007	0.008 ±0.001	0.01 ±0.001
α-Thujene	0.009 ±0.001^{ab}	0.008 ±0^{b}	0.008 ±0^{b}	0.009 ±0.001^{ab}	0.01 ±0.001^{a}	0.01 ±0^{a}
α-Fenchene	0.006 ±0	0.006 ±0	0.006 ±0	0.006 ±0	0.006 ±0	0.007 ±0.001
α-Humulene	0.152 ±0.013^{abc}	0.09 ±0.011^{c}	0.103 ±0.019^{bc}	0.142 ±0.017^{abc}	0.16 ±0.017^{a}	0.17 ±0.01^{ab}
Camphene	0.15 ±0.008^{ab}	0.126 ±0.007^{b}	0.129 ±0.007^{b}	0.147 ±0.004^{ab}	0.166 ±0.005^{a}	0.162 ±0.011^{a}
γ-Elemene	0.092 ±0.009^{bc}	0.041 ±0.006^{d}	0.046 ±0.01^{d}	0.062 ±0.006^{cd}	0.1 ±0.009^{ab}	0.129 ±0.006^{a}
Sabinene	0.011 ±0.003	0.011 ±0.001	0.02 ±0.005	0.024 ±0.006	0.012 ±0.004	0.017 ±0.002
α-Phellandrene	0.011 ±0.001	0.011 ±0.002	0.009 ±0.001	0.015 ±0.002	0.013 ±0.002	0.015 ±0.001

Terpene Compound (mg g^{-1})	HPS	Amber	Red	Rose	Purple	Blue
β-Phellandrene	0.075 ±0.016	0.073 ±0.007	0.079 ±0.006	0.097 ±0.009	0.106 ±0.019	0.105 ±0.015
1,8-Cineole	0.053 ±0.022	0.014 ±0.003	0.051 ±0.016	0.021 ±0.002	0.088 ±0.032	0.088 ±0.025
(Z)-β-Ocimene	0.014 ±0.001^{ab}	0.01 ±0.001^{b}	0.011 ±0.001^{ab}	0.013 ±0.001^{ab}	0.017 ±0.001^{a}	0.015 ±0.002^{ab}
γ-Terpinene	0.013 ±0.001^{ab}	0.01 ±0.001^{c}	0.011 ±0.001^{bc}	0.013 ±0^{ab}	0.015 ±0.001^{a}	0.014 ±0.001^{ab}
cis-Sabinene hydrate	0.033 ±0.004	0.026 ±0.006	0.026 ±0.005	0.03 ±0.002	0.032 ±0.005	0.033 ±0.007
Fenchone	0.066 ±0.004	0.054 ±0.006	0.055 ±0.008	0.064 ±0.005	0.074 ±0.003	0.067 ±0.002
Terpinolene	0.039 ±0.003^{ab}	0.031 ±0.003^{b}	0.035 ±0.004^{ab}	0.04 ±0.002^{ab}	0.046 ±0.002^{a}	0.037 ±0.004^{ab}
trans-Sabinene hydrate	0.01 ±0.001	0.009 ±0.001	0.01 ±0.001	0.01 ±0.001	0.012 ±0.001	0.012 ±0.001
cis-Pinene hydrate	0.07 ±0.006	0.049 ±0.008	0.055 ±0.009	0.063 ±0.007	0.076 ±0.005	0.077 ±0.004
Camphene hydrate	0.027 ±0.002	0.02 ±0.002	0.023 ±0.003	0.025 ±0.002	0.03 ±0.002	0.033 ±0.008
Ipsdienol	0.039 ±0.002	0.035 ±0.001	0.04 ±0.004	0.032 ±0.006	0.04 ±0.001	0.035 ±0.002
Terpinen-4-ol	0.015 ±0.001^{b}	0.013 ±0.001^{b}	0.014 ±0.002^{b}	0.016 ±0.001^{ab}	0.021 ±0.003^{ab}	0.021 ±0.001^{a}
Geraniol	0.015 ±0.002	0.012 ±0.002	0.012 ±0.002	0.014 ±0.001	0.018 ±0.001	0.015 ±0.001

Terpene Compound (mg g^{-1})	HPS	Amber	Red	Rose	Purple	Blue
α-Cubebene	0.008 ±0	0.007 ±0	0.008 ±0.001	0.008 ±0.001	0.009 ±0.001	0.01 ±0.001
α-Ylangene	0.008 ±0.001	0.006 ±0	0.006 ±0.001	0.009 ±0.001	0.009 ±0.001	0.009 ±0
Hexyl hexanoate	0.06 ±0.008	0.058 ±0.005	0.062 ±0.011	0.038 ±0.009	0.045 ±0.003	0.054 ±0.003
trans-α-Bergamotene	0.053 ±0.013	0.022 ±0.002	0.028 ±0.004	0.03 ±0.002	0.034 ±0.005	0.026 ±0.004
allo-Aromadendrene	0.006 ±0.001	0.005 ±0	0.006 ±0.001	0.006 ±0	0.006 ±0	0.005 ±0.001
(E)-β-Farnesene	0.017 ±0.002	0.011 ±0.001	0.013 ±0.002	0.015 ±0.002	0.017 ±0.003	0.014 ±0.003
β-Selinene	0.078 ±0.005[a]	0.044 ±0.003[b]	0.06 ±0.007[ab]	0.046 ±0.012[ab]	0.083 ±0.009[a]	0.093 ±0.006[a]
α-Selinene	0.068 ±0.005	0.056 ±0.01	0.057 ±0.008	0.07 ±0.005	0.096 ±0.016	0.066 ±0.022
β-Bisabolene	0.014 ±0.002	0.012 ±0.002	0.016 ±0.003	0.014 ±0.002	0.014 ±0.004	0.018 ±0.003
(3E,6E)-α-Farnesene	0.078 ±0.013[ab]	0.048 ±0.007[b]	0.052 ±0.01[ab]	0.055 ±0.017[ab]	0.083 ±0.02[ab]	0.116 ±0.02[a]
Spirovetiva-1(10),7(11)-diene	0.054 ±0.007	0.037 ±0.005	0.034 ±0.008	0.048 ±0.011	0.045 ±0.004	0.065 ±0.009
(E)-α-Bisabolene	0.087 ±0.005[a]	0.057 ±0.004[b]	0.076 ±0.01[ab]	0.082 ±0.006[ab]	0.085 ±0.008[ab]	0.08 ±0.005[ab]
Eudesma-5,7(11)-diene	0.039 ±0.003[ab]	0.028 ±0.002[b]	0.03 ±0.003[b]	0.037 ±0.003[ab]	0.045 ±0.005[a]	0.045 ±0.002[a]

Terpene Compound (mg g^{-1})	HPS	Amber	Red	Rose	Purple	Blue
(E)-Nerolidol	0.058 ±0.006^{ab}	0.041 ±0.004^{b}	0.045 ±0.006^{ab}	0.055 ±0.006^{ab}	0.066 ±0.008^{ab}	0.072 ±0.006^{a}
Caryophyllene oxide	0.021 ±0.003	0.019 ±0.008	0.013 ±0.004	0.045 ±0.017	0.07 ±0.027	0.087 ±0.027
Humulene epoxide II	0.041 ±0.005^{ab}	0.02 ±0.002^{c}	0.022 ±0.005^{bc}	0.031 ±0.005^{abc}	0.047 ±0.007^{a}	0.045 ±0.003^{a}
Juniper camphor	0.044 ±0.005^{abc}	0.025 ±0.003^{c}	0.031 ±0.006^{bc}	0.038 ±0.005^{abc}	0.049 ±0.007^{ab}	0.056 ±0.003^{a}
Aromadendrane-4,10-diol	0.018 ±0.002	0.013 ±0.002	0.021 ±0.002	0.02 ±0.002	0.021 ±0.002	0.017 ±0.003
meta-Camphorene	0.021 ±0.003^{ab}	0.013 ±0.002^{b}	0.013 ±0.003^{ab}	0.017 ±0.003^{ab}	0.024 ±0.003^{ab}	0.025 ±0.001^{a}
Phytol	0.127 ±0.017	0.138 ±0.029	0.193 ±0.031	0.18 ±0.035	0.259 ±0.061	0.185 ±0.031
δ-Guaiene	0.018 ±0.002	0.034 ±0	0.019 ±0.003	0.024 ±0.005	0.029 ±0.006	0.026 ±0.002
Eremophila-1(10),7(11)-diene	0.041 ±0.003	0.035 ±0.002	0.03 ±0.005	0.046 ±0.005	0.053 ±0.008	0.058 ±0.012
Cryptomeridiol	0.028 ±0.009	0.007 ±0.001	0.036 ±0.003	0.035 ±0.005	0.031 ±0.012	0.039 ±0.009
para-Cymene	0.006 ±0.001	0.005 ±0.001	0.006 ±0	0.007 ±0.001	0.006 ±0.001	0.006 ±0.001
α-Terpinene	0.007 ±0.001	0.009 ±0.002	0.007 ±0.001	0.009 ±0.002	0.008 ±0.002	0.007 ±0.001
Valencene	0.018 ±0.003	ND	0.014 ±0.002	0.017 ±0.006	0.034 ±0.014	0.097 ±0.002

Terpene Compound (mg g^{-1})	HPS	Amber	Red	Rose	Purple	Blue
α-Santalene	0.012 ±0.002	0.007 ±0.003	0.012 ±0.001	0.014 ±0	0.01 ±0.003	0.082 ±0.062
(4Z)-Decenol	0.016 ±0.003	0.013 ±0.002	ND	0.023 ±0.001	0.019 ±0.001	0.019 ±0.001
Selin-6-en-4α-ol	0.019 ±0.006	0.005 ±0.001	0.01 ±0.002	0.011 ±0.001	0.011 ±0.002	0.014 ±0.002
α-Guaiene	0.003 ±0.001	0.002 ±0	0.016 ±0.002	ND	0.018 ±0.008	0.019 ±0.006
Hashishene	0.002 ±0.001	0.002 ±0.001	0.003 ±0	ND	0.006 ±0.001	0.004 ±0
Decanol	0.005 ±0.001^{ab}	0.004 ±0^{b}	0.007 ±0^{a}	ND	0.004 ±0.001^{b}	0.003 ±0^{b}
Epoxyterpinolene	ND	ND	ND	ND	ND	0.007 ±0.001
Δ3-Carene	0.004 ±0	ND	0.003 ±0	0.004 ±0	ND	0.003 ±0.001
para-Cymenene	0.006 ±0.001	ND	ND	ND	0.004 ±0.001	0.001 ±0
Selin-6-en-4α-ol isomer	ND	ND	ND	ND	ND	0.043 ±0.004
Caryophylla-3,8(13)-dien-5β-ol	0.017 ±0.003	0.004 ±0	0.012 ±0.002	0.01 ±0.001	0.012 ±0.002	0.018 ±0.007
(2E,6E)-Farnesol	0.041 ±0.006^{ab}	0.022 ±0.007^{ab}	0.047 ±0.004^{ab}	0.015 ±0^{b}	0.024 ±0.001^{ab}	0.045 ±0^{a}
Hexyl butyrate	ND	ND	ND	ND	ND	0.004 ±0.001
para-Cymen-8-ol	ND	ND	0.003 ±0	ND	ND	ND
